# Detection of PIK3CA hotspot mutations in canine mammary tumors using droplet digital PCR: tissue validation and liquid biopsy feasibility

**DOI:** 10.1038/s41598-024-76820-0

**Published:** 2024-10-26

**Authors:** Byung-Joon Seung, Jung-Hyang Sur

**Affiliations:** 1 Department of Veterinary Pathology, College of Veterinary Medicine, Konkuk University, Seoul, 05029 South Korea; 2 Present Address: Department of Pathobiology, College of Veterinary Medicine, University of Illinois at Urbana-Champaign, Urbana, IL 61802 USA; 3 Present Address: Komipharm International Co., Ltd., Siheung-si, Gyonggi-do 15094 South Korea

**Keywords:** Canine mammary tumors, *PIK3CA* mutations, Droplet digital PCR, Liquid biopsy, Circulating tumor DNA, Cancer, Tumour biomarkers, Breast cancer

## Abstract

Domestic dogs (*Canis lupus familiaris*) serve as valuable translational models for human cancer research due to their biological similarities. Canine mammary tumors (CMTs), frequently diagnosed in female dogs, share various characteristics with human breast cancers. This study investigates the *PIK3CA* (H1047R) mutation in CMTs using droplet digital PCR (ddPCR) and explores the potential of liquid biopsy for non-invasive detection. We analyzed 80 formalin-fixed, paraffin-embedded (FFPE) CMT tissue samples and compared ddPCR results with next-generation sequencing (NGS) data, achieving high concordance. Plasma and serum samples were also assessed for mutation concordance with tissue results. Our findings indicate a higher frequency of the *PIK3CA* (H1047R) mutations in benign and grade I malignant CMTs compared to more aggressive malignancies. The ddPCR assay demonstrated high sensitivity and specificity, with plasma testing showing 78.6% sensitivity and 87.5% specificity, and serum testing showing 66.7% sensitivity and 90.0% specificity. These results highlight the viability of liquid biopsy as a minimally invasive method for monitoring *PIK3CA* mutations in canine patients. The study suggests that liquid biopsy techniques hold significant promise for improving the early detection and monitoring of canine cancers, warranting further research to refine these methods and explore their applications in canine cancer diagnostics and treatment.

**Supplementary Information:**

The online version contains supplementary material available at 10.1038/s41598-024-76820-0.

## Introduction

Domestic dogs (*Canis lupus familiaris*) serve as exceptional models of human cancer research^1^. Unlike genetically engineered mouse models, canine models offer a closer resemblance to human biology, as they naturally develop tumors within the context of an intact immune system in a common living environment^2^. Among canine cancers, canine mammary tumors (CMTs) are the most frequently diagnosed cancers in female dogs, mirroring human breast cancers in women^3,4^. CMTs share various similarities with human breast cancers, including etiology, pathogenesis, and histological classification^5^. Therefore, studying CMTs is crucial for gaining insights into molecular events leading to carcinogenesis, benefiting both human and veterinary oncology.

The emergence of non-invasive tests, such as liquid biopsy, has garnered increasing attention in both human and veterinary oncology due to their ability to provide accessible and minimally invasive sampling^6,7^. Liquid biopsy techniques can capture various entities within the blood, including cell-free DNA (cfDNA) and circulating tumor DNA (ctDNA)^6^. Recent advancements in both next-generation sequencing (NGS) technologies and droplet digital PCR (ddPCR) have enabled the detection of cancer-specific somatic variations in blood samples, offering promise for the development of minimally invasive diagnostic tools in oncology^8^.

The phosphatidylinositol-4,5-bisphosphate 3-kinase catalytic subunit alpha **(***PIK3CA*) gene, encoding the p110α subunit of PI3Kα, plays a crucial role in the PI3K/AKT signaling pathway by phosphorylating phosphatidylinositol 4,5-bisphosphate (PIP2) to produce phosphatidylinositol 3,4,5-trisphosphate (PIP3)^9^. The *PIK3CA *gene stands as one of the most frequently mutated genes in human breast cancer^10,11^. Approximately 80% of the *PIK3CA *mutations cluster within three hotspots in the coding sequence: E542K (~ 4% of human breast cancer) and E545K (~ 6%) within the helical domain; and H1047R (~ 15%) within the kinase domain of p110 α^12–14^. These hotspot mutations, such as E545K and H1047R, enhance the production of phosphatidylinositol (3,4,5)-trisphosphate [PI(3,4,5)P_3_] levels, leading to constitutive activation of the PI3K/AKT signaling pathway^15^. Consequently, these mutations induce oncogenic transformation by constitutive phosphorylation of AKT^16^, providing a compelling rationale for the development of PI3Kα inhibitors targeting these specific gene mutations^14^.

Recent advances in NGS have enabled the characterization of the molecular landscape of CMTs, revealing somatic mutations similar to those observed in human breast cancers. The initial frequency of the *PIK3CA *mutation in CMTs was first reported by Lee et al.^17^. Our previous study extends this work by examining a larger and clinically annotated cohort, confirming an overall mutation frequency of 43.1% and providing deeper insights into the clinical implications of these mutations^18^. Among the 91 *PIK3CA *missense mutations analyzed, 53 cases were identified as the H1047R mutation, constituting 58.2% of the total^18^. This prevalence suggests that CMTs harbor frequent mutations in the PI3K pathway, akin to human breast cancer^18,19^. Additionally, the higher mutation frequency of the *PIK3CA* gene in benign CMTs compared to malignant CMTs hints at a potential early oncogenic role of *PIK3CA *mutations in development of CMTs^18^. These observations underscore the importance of understanding *PIK3CA* mutations in the context CMTs and highlight their potential as therapeutic targets in veterinary oncology.

Given the growing significance of *PIK3CA* mutations in CMTs, our study aims to conduct a comprehensive analysis of *PIK3CA* mutations in CMTs using ddPCR across various sample types, including archived formalin-fixed paraffin-embedded (FFPE) tissue samples and blood samples including plasma and serum. Initially, we seek to validate the presence of the *PIK3CA* (H1047R) mutations in archived FFPE tissue samples, harnessing the sensitivity and specificity of ddPCR. Subsequently, we endeavor to corroborate our finding through comparison with results obtained from NGS analysis, thereby providing a robust assessment of the *PIK3CA* (H1047R) mutation status in CMTs. Furthermore, we aim to evaluate the association between the presence of the *PIK3CA* (H1047R) mutations and various clinical parameters, such as tumor grade and histological subtype. In addition, our study investigates the potential utility of circulating tumor DNA (ctDNA) as a liquid biopsy marker for CMT diagnosis. We aim to identify the recurrent *PIK3CA* (H1047R) mutations in ctDNA extracted from canine blood samples including plasma and serum, with the objective of establishing a minimally invasive diagnostic approach for CMTs. By analyzing ctDNA, we seek to elucidate whether the *PIK3CA* (H1047R) mutations can serve as reliable biomarkers for cancer detection and monitoring in dogs, thereby offering insights into the potential of liquid biopsy marker in CMTs.

## Results

### Sample characteristics

In this study, FFPE tissue samples from 80 individual dogs diagnosed with CMTs were utilized. Each sample originated from a different dog, ensuring unique representation across the study. Additionally, matched plasma samples were available from 38 dogs, and matched serum samples from another 42 dogs. Notably, blood samples were collected preoperatively as part of routine diagnostic procedures, generally close to the time of surgical intervention. The most represented breed of dogs in our study was Maltese dogs (20/80, 25%). The median onset age of CMT in our cohorts is approximately 11.00 years. Histologically, the tumors were classified as benign (*n* = 12), and malignant (*n* = 68). Among malignant CMTs, histological grades included grade I (*n* = 41), grade II (*n* = 13), and grade III (*n* = 14). Lymphatic invasion was evident in seven cases of malignant CMTs. Further details regarding sample characteristics are provided in Supplementary Table 1.

### ***PIK3CA ***(H1047R) mutation in FFPE tumor samples

**Fig. 1:**
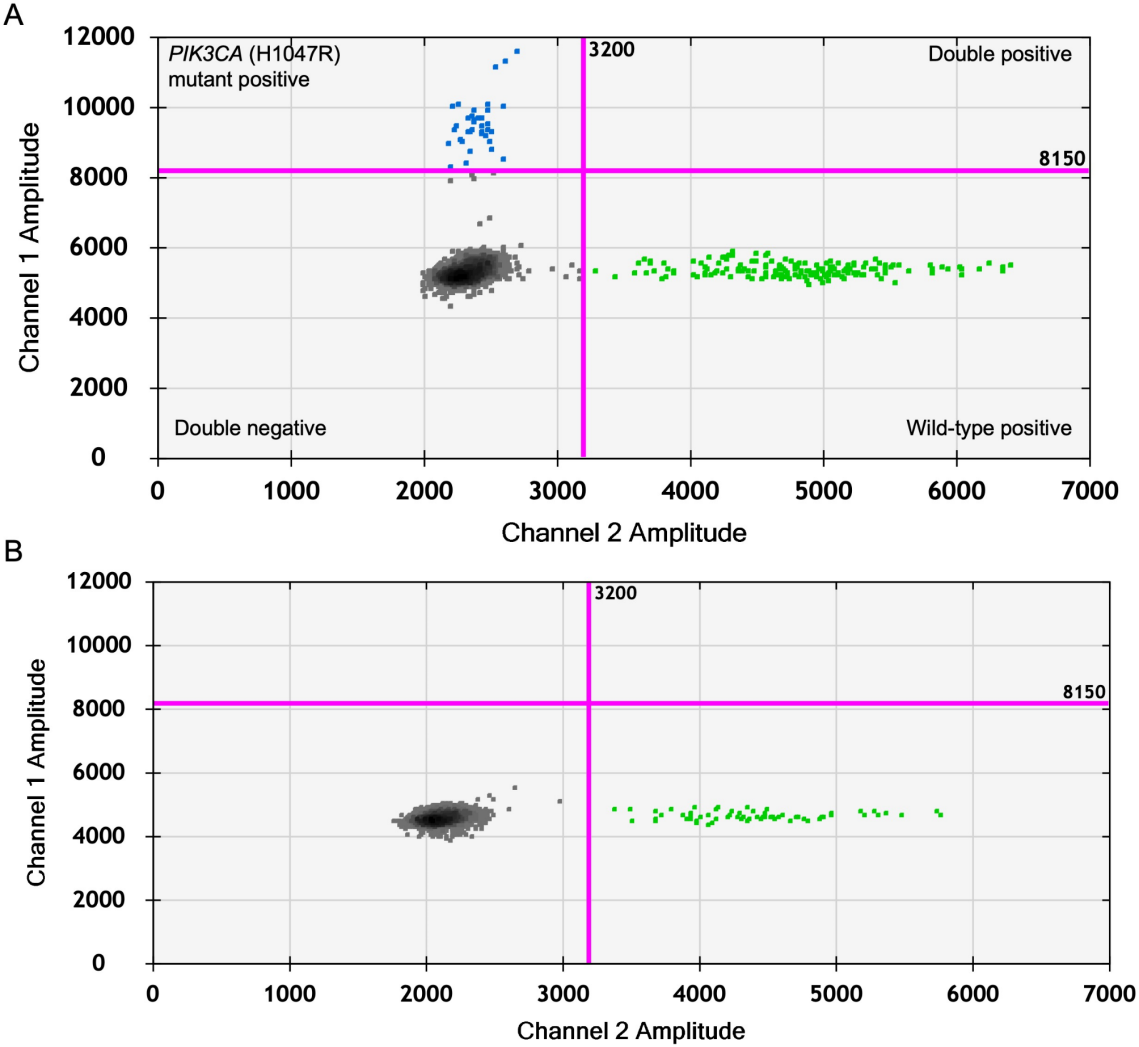
Analysis of the *PIK3CA* (H1047R) mutation by droplet digital PCR assay in FFPE tissue samples. Representative 2-D plots of ddPCR for the *PIK3CA* (H1047R) mutation positive droplets (blue) and the *PIK3CA* wild-type droplets (green) in CMT tissue sample (Sample No. 6) (**A**) and a non-neoplastic tissue sample (**B**). Each point represents a droplet, and those above the pink threshold line are classified as positive.

In our study, we investigated *PIK3CA* somatic recurrent mutations in FFPE tissue samples obtained from dogs diagnosed with CMTs. Given the prevalence of H1047R amino-acid residue mutations in the *PIK3CA *gene according to the previous article^18^, we performed the ddPCR assays specifically targeting the hotspot mutations (H1047R) in the *PIK3CA* gene in CMTs (Fig. 1 and Supplementary Figure S1). Our findings revealed that the *PIK3CA* (H1047R) mutations were present in 26 out of 80 (32.5%) of the CMT FFPE samples. Among the 26 FFPE samples with detectable the *PIK3CA* (H1047R) mutation, the fractional abundance ranged from 1.08% to 80.07% with a median of 21.87% (Supplementary Table 1).

In benign CMT cases, the *PIK3CA* (H1047R) mutations were detected in 5 out of 12 cases (41.7%). Regarding malignant CMT cases, the presence of the *PIK3CA* (H1047R) mutations varied according to histological grade (Grades I: 17 out of 41 cases, 41.7%; Grade II: 2 out of 13 cases, 15.4%; Grade III 2 out of 14 cases, 14.3%) (Table 1). Notably, the presence of the *PIK3CA* (H1047R) mutation was more prominent in benign CMT and grade I malignant CMT cases (22 out of 53 cases, 41.5%) compared to grade II and III malignant CMT cases (4 out of 27 cases, 14.8%) (Chi-square test, *P* = 0.016) (Table 1). However, no significant associations were found between the *PIK3CA* (H1047R) mutation status and other clinical parameters.

**Table 1:** Correlation between presence of the *PIK3CA* (H1047R) mutation and other clinical parameters.

	Number of samples (%)		
	*PIK3CA* (H1047R)		*P* value
	Positive	Negative	
Neuter status			
Intact female (*n* = 49)	19 (38.8%)	30 (61.2%)	0.170^a^
Spayed female (*n* = 26)	6 (23.1%)	20 (76.9%)	
Histological subtype			
Simple type (*n* = 40)	13 (32.5%)	27 (67.5%)	0.719^a^
Complex type (*n* = 30)	9 (30%)	21 (70%)	
Mixed type (*n* = 9)	4 (44.4%)	5 (55.6%)	
Histological grade			
Grade I (*n* = 41)	17 (41.5%)	24 (58.5%)	0.079^b^
Grade II (*n* = 13)	2 (15.4%)	11 (84.6%)	
Grade III (*n* = 14)	2 (14.3%)	12 (85.7%)	
Histological grade			
Benign and Grade I (*n* = 53)	22 (41.5%)	31 (58.5%)	**0.016** ^a^
Grade II and Grade III (*n* = 27)	4 (14.8%)	23 (85.2%)	
Lymphatic invasion			
Absent (*n* = 73)	25 (34.2%)	48 (65.8%)	0.418^b^
Present (*n* = 7)	1 (14.3%)	6 (85.7%)	

^a^Chi-square test.

^b^Fisher’s exact test.

Forty-two dogs with CMT cases, for which the *PIK3CA*(H1047R) mutation was confirmed by NGS in the previous study^18^, were included in this study. In that study, each of these 42 tumor tissue samples was bisected upon collection: one half was frozen for NGS analysis, and the other half was processed into FFPE blocks for histological examination. For ddPCR analysis in the current study, DNA was extracted from the FFPE sections. Therefore, although both analyses were performed on material from the same tumors, NGS used DNA from frozen tissue while ddPCR used DNA from FFPE tissue. There was close agreement between detection of the *PIK3CA* (H1047R) mutation by ddPCR and NGS, with Cohen’s κ coefficient = 0.883 (95% CI, 0.726-1.000), with concordance rate 95.2% (40/42 cases) (Table 2). Additionally, a Spearman’s correlation analysis revealed a strong positive correlation between the VAF values measured by ddPCR and NGS (ρ = 0.911, *p* < 0.001), further supporting the consistency between the two methods in quantifying the mutation burden.

**Table 2:** Comparison of the *PIK3CA* (H1047R) mutation detected in tumor tissue samples of canine mammary tumors by ddPCR versus the mutation status detected in paired tumor tissue by NGS method.

*PIK3CA* (H1047R) mutation	Number of samples		
	NGS method		Total
	Positive	Negative	
ddPCR method			
Positive	11	1	12
Negative	1	29	30
Total	12	30	42

### *PIK3CA* (H1047R) mutation in blood samples

**Fig. 2:**
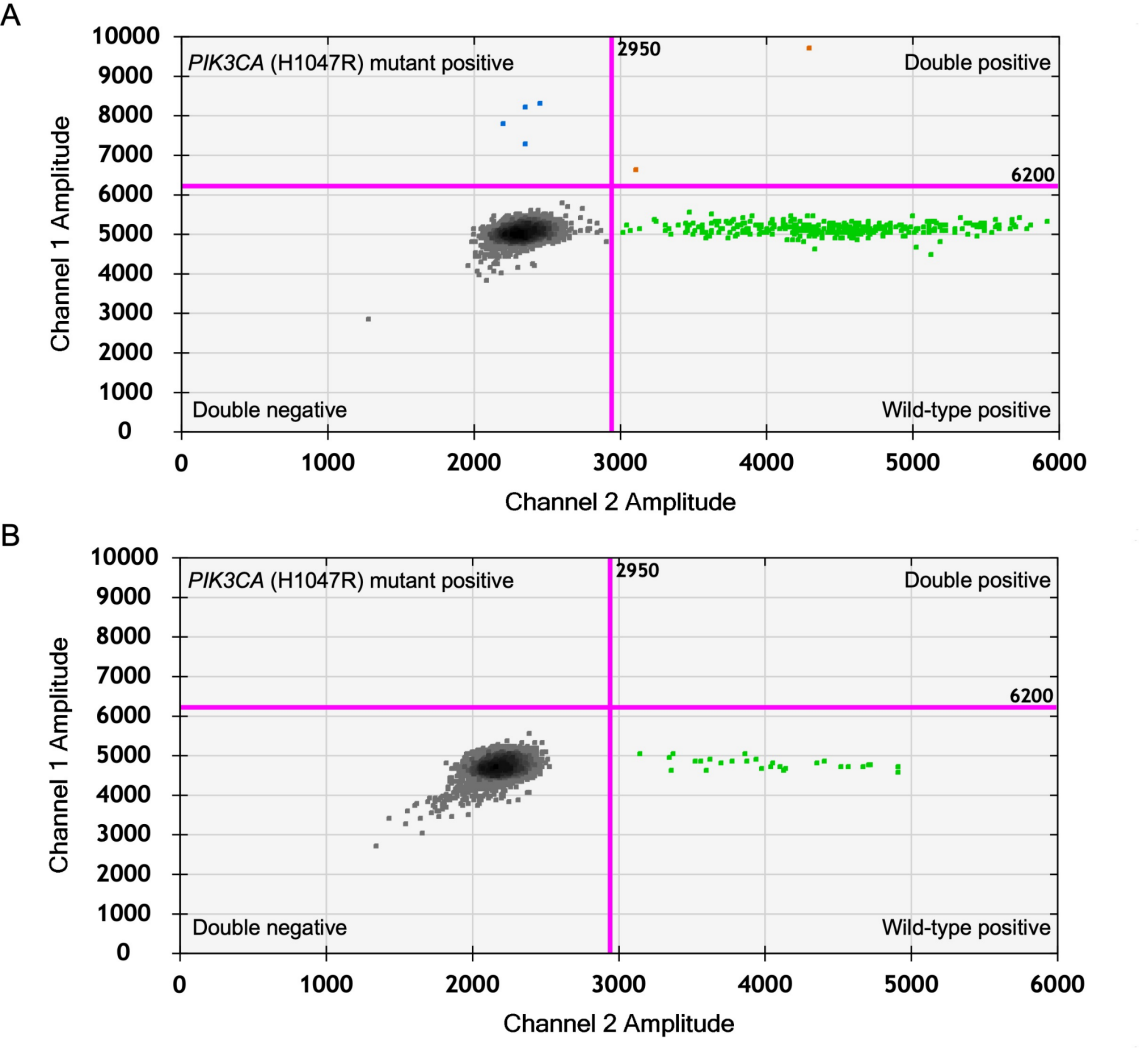
Analysis of the *PIK3CA* (H1047R) mutation in blood samples using liquid biopsy with droplet digital PCR assay. Representative 2-D plots of ddPCR for the *PIK3CA* (H1047R) mutation positive droplets (blue) and the *PIK3CA* wild-type droplets (green) from the serum of a CMT patient (Sample No. 6) (**A**) and from a healthy control (**B**). Each point represents a droplet, and those above the pink threshold line are classified as positive.

We investigated the plasma samples from 38 dogs with CMT and serum samples from 42 dogs with CMT, comparing them with matched-pair FFPE tumor tissue samples through ddPCR assay (Fig. 2 and Supplementary Fig. 2). Our results revealed that the *PIK3CA* (H1047R) mutations were found in 14 out of 38 cases (36.84%) of plasma samples from dogs with CMT. The fractional abundance of the *PIK3CA* (H1047R) mutation in the plasma samples where the mutation was detected ranged from 0.011% to 7.273%, with a median of 0.399% (Supplementary Table 1). The concordance rate between the detection of the *PIK3CA* (H1047R) mutation in tumor tissue and plasma samples was 84.2% (32 out of 38 cases), with a Cohen’s κ coefficient of 0.661 (95% CI, 0.412–0.910). The sensitivity and specificity for plasma testing of the *PIK3CA* (H1047R) mutation by ddPCR were 78.6% (11 out of 14 cases; 95% CI, 0.488–0.942) and 87.5% (21 out of 24 cases; 95% CI, 0.665–0.967), respectively. The positive predictive value was 78.6% (11 out of 14 cases; 95% CI, 0.488–0.943) (Table 3).

**Table 3:** Comparison of the *PIK3CA* (H1047R) mutation detected in serum and plasma samples from dogs with canine mammary tumors by ddPCR versus the mutation status detected in paired FFPE tumor tissue samples.

*PIK3CA* (H1047) mutation status (ddPCR)	FFPE tumor tissue (Number of samples)		Sensitivity (%)	Specificity (%)	Positive predictive value (%)	Concordance (%)	Cohen’s κ coefficient (95% CI)
	Positive	Negative					
Serum (*n* = 42)							
Positive	8	3	66.7	90.0	72.7	83.3	0.581 (0.305–0.857)
Negative	4	27					
Plasma (*n* = 38)							
Positive	11	3	78.6	87.5	78.6	84.2	0.661 (0.412–0.910)
Negative	3	21					
Total blood (*n* = 80)							
Positive	19	6	73.1	88.9	76.0	83.8	0.626 (0.442–0.810)
Negative	7	48					

In cases of serum samples from dogs with CMT, the *PIK3CA* (H1047R) mutations were found in 11 out of 42 cases (26.19%). The fractional abundance of the *PIK3CA* (H1047R) mutation in these serum samples where the mutation was detected ranged from 0.066% to 5.512%, with a median of 0.756% (Supplementary Table 1). The concordance rate between the detection of the *PIK3CA* (H1047R) mutation in tumor tissue and serum samples was 83.3% (35 out of 42 cases), with a Cohen’s κ coefficient of 0.581 (95% CI, 0.305–0.857). The sensitivity and specificity for serum testing of the *PIK3CA* (H1047R) mutation by ddPCR were 66.7% (8 out of 12 cases; 95% CI, 0.354–0.887) and 90.0% (27 out of 30 cases; 95% CI, 0.723–0.974), respectively. The positive predictive value was 72.7% (8 out of 11 cases; 95% CI, 0.393–0.927) (Table 3).

Overall, the concordance rate between blood sample and tumor tissue testing was 83.8% (67 out of 80 cases), with a Cohen’s κ coefficient of 0.626 (95% CI, 0.442–0.810). The sensitivity and specificity for blood testing of the *PIK3CA* (H1047R) mutation by ddPCR were 73.1% (19 out of 26 cases; 95% CI, 0.519–0.876) and 88.9% (48 out of 54 cases; 95% CI, 0.767–0.954), respectively. The positive predictive value was 76.0% (19 out of 25 cases; 95% CI, 0.545–0.898) (Table 3).

## Discussion

In this study, we examined the frequency of the *PIK3CA* (H1047R) mutation in 80 CMT tissue samples using the ddPCR rare mutation detection assay. We identified the *PIK3CA *(H1047R) mutations in 32.5% (26/80) of CMT FFPE samples, a significant increase compared to our previous study^20^, which detected this mutation in 14.3% (10/70) of tumor samples via Sanger sequencing. This discrepancy likely stems from the enhanced sensitivity of ddPCR, which offers improved detection capabilities for somatic mutations through sample partitioning, increasing mutation concentration in each droplet^21,22^. Additionally, the specificity of ddPCR is particularly advantageous for analyzing FFPE samples, where DNA degradation is common^21^.

Interestingly, our data indicated a higher frequency of the *PIK3CA* (H1047R) mutations in benign and grade I malignant CMTs (41.5%) compared to more aggressive grade II and III malignancies (14.8%). This pattern suggests that *PIK3CA *mutations might play an early oncogenic role in CMT development, aligning with previous studies reporting higher mutation rates in benign CMTs than in malignant ones^18,23^. In human breast cancers, *PIK3CA *mutations are more frequent in hormone receptor-positive/HER2-negative tumors (around 42%), typically low-grade, and less common in higher-grade triple-negative breast cancers (approximately 16%)^24^. A tumor-initiating role for the *PIK3CA *(H1047R) variant has been demonstrated in mouse breast tumor models, and functionally, this mutation has been shown to increase PI3K enzyme activity in vivo^25,26^. This enhancement of enzyme activity promotes cell proliferation and survival signaling pathways, supporting the idea that the *PIK3CA* mutation contributes to early oncogenic signaling.

Regarding histological subtypes, our studies included both benign and malignant CMTs, including complex and mixed types being common in CMTs due to their heterogeneity. In contrast, human breast cancers are predominantly of the simple type, such as invasive ductal carcinoma (IDC) and invasive lobular carcinoma (ILC), and the TCGA dataset focuses primarily on these malignant cases^10^. Complex and mixed subtypes, particularly those characterized by myoepithelial cell proliferation or metaplastic changes involving bone and cartilage formation, are exceedingly rare in human breast cancers^27^. This difference is important when comparing mutation frequencies across species, as the prevalence of certain histological subtypes may influence the observed mutation rates.

The ddPCR results showed high concordance with available NGS data^18^, achieving a concordance rate of 95.2% (Cohen’s κ coefficient = 0.883). Moreover, the strong positive correlation between the VAF values measured by ddPCR and NGS (Spearman’s ρ = 0.911, *p* < 0.001) further supports the consistency between the two methods in quantifying the mutation burden. The high concordance demonstrates that the sensitive ddPCR method not only effectively validates the accuracy of NGS results for detecting *PIK3CA* mutations but also highlights its complementary role in ensuring robust and reliable mutation detection.

In analyzing the 42 matched samples, we observed discrepancies in two cases that underscore the complexities inherent in mutation detection across different platforms. In the case of Sample No. 23, where the mutation was identified by NGS but not by ddPCR, and Sample No. 8, which demonstrated the inverse pattern, a number of factors may be responsible for these outcomes. Such discrepancies may be attributed to the potential degradation of DNA, which can impede the accuracy of ddPCR^28^. Furthermore, the discrepancies observed may also be attributed to differences in the DNA source, with frozen tissue being used for NGS and FFPE sections for ddPCR. Although the NGS and ddPCR were conducted on the same tumor samples, the disparate methods of tissue processing and DNA extraction may have influenced the mutation detection outcomes. Furthermore, the intrinsic sensitivity of ddPCR enables the detection of mutations at low fractional abundances that may be overlooked by NGS, as evidenced by Sample No. 8. These instances underscore the challenges of using molecular diagnostics and highlight the importance of integrating multiple methods to ensure robust mutation detection, particularly in complex cases where tumor biology may affect the consistency of results across different assays.

We also explored the potential application of liquid biopsy for detecting *PIK3CA* mutations in CMTs by analyzing blood samples from the same patients. The concordance rates between tumor tissue and plasma or serum samples were 84.2% and 83.3%, respectively. Plasma testing showed 78.6% sensitivity and 87.5% specificity, while serum testing showed 66.7% sensitivity and 90.0% specificity. These findings highlight liquid biopsy as a viable, minimally invasive method for monitoring *PIK3CA* mutations, which could significantly benefit early detection and treatment response monitoring in canine patients.

However, some discrepancies were noted between the blood and tissue results. For instance, in Sample No. 8, the fractional abundance of the *PIK3CA *(H1047R) mutation was higher in serum than in the corresponding tissue. This could be due to several factors, including tumor heterogeneity or the differential shedding rates of ctDNA into the bloodstream^29^. Moreover, lower wild-type allele counts in the serum sample may have resulted in an inflated mutation fraction, highlighting the challenges in interpreting liquid biopsy results when droplet counts are variable. Similarly, there were instances where the mutation was detected in serum/plasma but not in the corresponding tissue sample. This might reflect sampling bias due to tumor heterogeneity, where the biopsy may not have captured all tumor clones. Additionally, given that canine mammary tumors often arise as multiple lesions^30^, it is possible that the mutation detected in the blood originated from another, unsampled tumor within the same dog. These findings underscore the value of liquid biopsy in complementing tissue biopsy, especially in cases where tissue sampling might not fully represent the entire tumor’s mutational profile.

Recent studies have increasingly highlighted the growing interest and effectiveness of liquid biopsy techniques in detecting various biomarkers in canine cancers. For instance, ddPCR has been effectively used to detect ctDNA in various canine tumors^31^. Additionally, NGS-based liquid biopsy tests have demonstrated high specificity (98.5%) and moderate sensitivity (54.7%) for multi-cancer detection, underscoring their potential for early cancer detection in dogs^7^. While these methods are promising, our study uniquely focuses on the *PIK3CA* (H1047R) mutation, a somatic alteration we previously identified in CMTs and have now validated in tissue using ddPCR. This study further explores the potential of liquid biopsy for early detection of this specific mutation, aiming to enhance the precision of diagnostic approaches and facilitate targeted therapies in veterinary practice.

While our study provides significant insights, it has several limitations. The retrospective nature of the study may introduce selection bias and focusing on a single hotspot mutation (H1047R) in the *PIK3CA *gene potentially overlooks other relevant mutations that could contribute to CMT pathogenesis. In addition, although blood samples were collected preoperatively, the exact intervals between these procedures were not systematically documented. The sensitivity and specificity of results may be influenced by the variability in ctDNA shedding rates among different clinical progression which can affect detection efficacy^29^. Moreover, there was variability in the volume of plasma or serum used for cfDNA extraction, ranging from 200 to 1000 µL. This variation likely contributed to differences in cfDNA yield and mutation detection sensitivity across samples. While we aimed to maximize detection by using the maximum available volume, it is important to emphasize that this study was an exploratory investigation designed to assess the feasibility of liquid biopsy for *PIK3CA* mutation detection in a clinical setting, rather than to establish a standardized protocol. Future studies should aim to standardize not only the DNA input amounts but also critical parameters such as detection thresholds and evaluation criteria to ensure consistent sensitivity and enable more precise comparisons between cases. Standardizing these factors will be essential for developing robust protocols and advancing the clinical utility of liquid biopsy techniques.

Although mutation fractions in blood are often lower compared to tissue samples, our data indicate that in approximately 10% of cases (9/80), the mutation fraction in blood was higher than in the corresponding tissue sample. This variability may be attributed to tumor heterogeneity, differences in ctDNA shedding rates, or technical variations between sample types. Notably, our results, which range from 0.011% to 7.273%, are consistent with the detection sensitivities reported in the literature. While one study reported mutation fractions ranging from 0.01% to 2.99% ^32^, another study has observed higher mutation fractions ranging from 0.27% to 49.85% in plasma from breast cancer patients^33^. Understanding this variation is crucial for the effective use of liquid biopsy in early cancer detection, as ctDNA levels can vary widely among patients and disease stages, reflecting different tumor burdens and metabolic activities.

The field of veterinary oncology has yet to fully utilize the power of genomics for its precision medicine benefits. Our study contributes to this growing field by demonstrating the practical application of ddPCR in identifying clinically significant mutations, paving the way for more comprehensive genomic analyses in veterinary oncology. Given the success of PI3K inhibitors in treating human breast cancer^34^, similar therapeutic strategies could be explored for dogs. The treatment of canine cancer may benefit from the development of novel human cancer drugs that target shared oncogenic mutations, such as alpelisib for metastatic breast cancer with *PIK3CA* mutations^35^. This approach underscores the importance of integrating advanced genomic tools in veterinary practice to enhance diagnostic accuracy and treatment outcomes. Future studies should focus on larger cohorts, including diverse breeds and stages of CMTs, and further explore the therapeutic potential of PI3K inhibitors in canine patients, paving the way for personalized medicine in veterinary oncology.

## Methods

### Study cohort and sample collection

The study was conducted retrospectively on 80 dogs diagnosed with CMTs, for which matched-pair of archived tissue and preoperative blood samples were available. These samples were procured during routine diagnostic procedures from privately owned dogs via private veterinary clinics, with any residual samples being retrospectively utilized in this study. Notably, 42 of these cases had previously undergone NGS analysis in our separate study^18,36^. For these 42 cases, tumor tissue samples were bisected upon collection, with one half being frozen for NGS analysis and the other half processed into FFPE blocks for histological examination. The sampling protocol adhered to the guidelines of the Institutional Animal Care and Use Committee of Konkuk University (KU16106, KU17162, and KU18168) as applicable.

For histopathological diagnosis, tissue samples were fixed in 10% neutral buffered formalin, processed routinely, and embedded in paraffin wax. Blood samples, primarily collected before surgery, were collected in BD Vacutainer^®^ EDTA tubes (Becton Dickinson, NJ, USA) or BD Vacutainer^®^ SST tubes (Becton Dickinson). Following collection, the tubes were gently inverted and centrifuged at 1,500 × *g* for 15 min. The resulting plasma or serum samples were then aliquoted into cryovials and stored at − 80 °C.

### Histopathology

For histopathological examination, 4-µm-thick sections were prepared from formalin-fixed paraffin- embedded (FFPE) blocks and stained with hematoxylin and eosin (H&E). Diagnosis was conducted by two pathologists (B-J Seung and J-H Sur), and the histological subtype of each sample was determined according to 2011 classification criteria for CMTs^37^. In cases where the subtype was ambiguous (e.g., simple adenoma versus simple carcinoma [Grade I], or benign mixed tumor versus carcinoma arising in a benign mixed tumor [Grade I] or complex adenoma versus complex carcinoma [Grade I]), malignancy was determined as outlined by Rasotto et al.^38^. Histological grade was assessed based on the criteria described by Peña et al.^39^. Lymphatic invasion, defined as infiltration of tumor cells in lymphatic vessel, was evaluated.

### DNA extraction

Each FFPE block was accompanied by a corresponding H&E-stained slide to guide the selective removal of adjacent normal tissue, ensuring an enrichment of tumor cells. Manual macrodissection was performed on eight 10 μm sections per sample, adjacent to those used for histopathological diagnosis, to precisely isolate tumor-rich areas. DNA was then extracted using the QIAamp DNA FFPE Tissue Kit (Qiagen, Hilden, Germany), following the manufacturer’s protocol.

For stored plasma and serum samples, aliquots were thawed, and a second centrifugation at 16,000 × *g* was carried out for 10 min prior to extraction. Circulating cfDNA was extracted from 200 to 1000 µL of plasma or serum using the Maxwell^®^ RSC ccfDNA Plasma Kit (Promega, Madison, WI, USA), as per the manufacturer’s instructions. Subsequently, DNA concentration was measured using QuantiFluor^®^ ONE dsDNA Dye kit (Promega, Madison, WI, USA) on a Quantus™ Fluorometer (Promega).

### Droplet digital PCR (ddPCR) protocol and data analysis

To detect *PIK3CA* somatic mutations in CMT samples, we conducted ddPCR assays using QX200 Droplet Digital PCR system (Bio-Rad, Hercules, CA, USA) following the manufacturer’s protocol. We targeted the hotspot mutations (H1047R) in the *PIK3CA *gene in CMTs, which had been identified as the most common mutation site in previous study^15^. The primers and probes were designed using Beacon Designer (ver 8.12; available at https://www.premierbiosoft.com/qOligo/Oligo.jsp?PID=1) and synthesized by Metabion Company (Metabion international AG, Semmelweisstr, Germany). Each 20 µL ddPCR reaction mixture comprised 10 µL of 2× ddPCR™ Supermix for Probes (Bio-Rad), 900 nM of each primer, 250 nM of each probes and 5.4 µL of eluted cfDNA as input (mean: 8.38 ng) or 5 ng of tumor DNA from tissue. Details of primers and probes are listed in Supplementary Table 2. 20 µL of each sample mixture was transferred into the middle well of droplet generator cartridges (Bio-Rad), followed by the addition of 70 µL of droplet generation oil for probes (Bio-Rad) to the lower well. Approximately 15,000 to 20,000 droplets were generated in the QX200 Droplet Generator (Bio-rad) and 40 µL of mixture was manually transferred into the wells of a 96-well plate. The plate was heat-sealed with pierceable foil heat seal (Bio-Rad) and loaded in a T100 thermal cycler (Bio-Rad). PCR was performed under the following conditions: 95 °C for 10 min, followed by 40 cycles of 94 °C for 30 s and 56 °C for 60 s, and a final extension step of 98 °C for 10 min, with a hold at 4 °C. A ramp rate of 2 °C/s was used for all steps. After PCR amplification, plates were transferred to the QX200™ Droplet Reader (Bio-Rad) for droplet analysis. The concentrations of the target sequences were calculated using the Poisson distribution using QuantaSoft software version 1.7.4 (Bio-Rad). Each experimental run included a positive control, comprising DNA extracted from FFPE tissue sampels previously confirmed to harbor the *PIK3CA* (H1047R) mutation by NGS, to verify the assay’s sensitivity. The fractional abundance in these positive controls ranged from 20% to 40%, representative of expected VAFs in tissue samples. Negative controls comprised a no-template control (NTC) to assess contamination and wild-type DNA (5ng) from non-neoplastic mammary tissue and blood samples (5.4 µL of eluted cfDNA from a 1,000 µL sample) from healthy dogs. Reactions with fewer than 8,000 droplets were considered invalid, and the experiment was repeated to ensure an adequate number of droplets.

After data acquisition, samples were analyzed using QuantaSoft software (Bio-Rad). Thresholds were manually defined for each assay following the guidelines from the Bio-Rad ddPCR application guide bulletin 6628 (Rare Mutation Detection Best Practices Guidelines [Internet]. Available: http://www.bio-rad.com/webroot/web/pdf/lsr/literature/Bulletin_6628.pdf, accessed in February 2022) and previous literature^31^. The estimation of the false-positive droplets was determined by conducting three wild-type control reactions using non-neoplastic tissue or blood sample from healthy dogs for each assay. The total number of detected mutation-positive droplets from three wild-type control reactions per each assay was considered false-positive and set as the threshold. Any sample with mutant droplet counts exceeding these thresholds was considered positive for the *PIK3CA* (H1047R) mutation. The frequency of the *PIK3CA* (H1047R) mutation in samples was determined by calculating the fraction of the mutant *PIK3CA* molecule concentration A (copies/µL) relative to the wild-type *PIK3CA* reference molecule concentration B (copies/µL) (fraction = A/(A + B)).

### Analyzing NGS data for variant allele frequency

For the 42 dogs with confirmed the *PIK3CA*(H1047R) mutation status by whole exome sequencing^18^, we compared the presence or absence of the *PIK3CA*(H1047R) mutation detected by ddPCR with those detected by whole-exome sequencing. While the results for all 42 dogs were obtained from the previous study^18^, we performed a re-analysis of the whole-exome sequencing data specifically for the 12 dogs that were positive for the *PIK3CA* (H1047R).

The whole-exome sequencing data for these 12 dogs were re-analyzed using CLC Genomics Workbench 23.0.5 software (Qiagen) to determine the depth of coverage and variant allele frequency (VAF) for the *PIK3CA*(H1047R) mutation. Briefly, FASTQ files were imported into the software, and reads were mapped to the CanFam3.1 reference genome using the “Map Reads to Reference” tool with the following parameters: mismatch cost of 2, insertion cost of 3, deletion cost of 3, length fraction of 0.8, and similarity fraction of 0.9. PCR duplicates were removed using the “Remove Duplicate Mapped Reads” tool. Local realignment around indels was performed using the “Local Realignment” tool to improve alignment accuracy. Variants were called using the “Low Frequency Variant Detection” tool with the following settings: required significance of 1%, minimum coverage of 10, minimum count of 2, minimum frequency of 1.0%, base quality filter enabled with a minimum central quality of 20 and read direction filters enabled. Quality control metrics were not re-assessed, as they were thoroughly evaluated in our previous study^18^. The depth of coverage and VAF at the *PIK3CA* (H1047R) position for the 12 positive samples were extracted from the variant calling results and are provided in Supplementary Table 1.

### Statistical analysis

The concordance of *PIK3CA* (H1047) mutation status between ddPCR and NGS results from tumor tissue was evaluated by Cohen’s κ test. Additionally, to evaluate the quantitative correlation between the VAF values from ddPCR and NGS, Spearman’s rank correlation analysis was performed. The sensitivity, specificity, positive predictive value, and concordance rate of the blood (plasma and serum) test (detected by ddPCR) was calculated by comparing with the matched tumor tissue result (detected by ddPCR). The result consistency between blood and tissue was assessed by Cohen’s κ test. Associations between the presence of the *PIK3CA* (H1047R) mutations and other clinical parameters were evaluated using Chi-square tests or Fisher’s exact tests. All statistical analyses were conducted using SPSS version 25 software for Windows (SPSS, Inc., Chicago, IL, USA). Results were considered statistically significant when *P* < 0.05.

## Electronic supplementary material

Below is the link to the electronic supplementary material.

Supplementary Material 1

Supplementary Material 2

Supplementary Material 3

## Data Availability

The raw whole exome sequencing datasets analyzed during the current study are available in Sequence Read Archive (SRA) with the accession numbers SRP159481. All other data supporting the findings of this study are available within the manuscript and its Supplementary information files.
