# Supplementary material for: Detection of PIK3CA hotspot mutations in canine mammary tumors using droplet digital PCR: tissue validation and liquid biopsy feasibility
Source: Sci Rep. 2024 Oct 26;14:25587. doi: 10.1038/s41598-024-76820-0 (PMC11512996; doi:10.1038/s41598-024-76820-0)
Supplement: Supplementary file 2 — Supplementary Material 2 [file 41598_2024_76820_MOESM2_ESM.docx]

**Supplementary Table 2**

**Details of the primers and probes for droplet digital PCR.**

| Product | Sequence (5' $\to$ 3') | 5' Reporter dye | 3' Quencher dye |
| --- | --- | --- | --- |
| Primer, forward | AGAGGCTTTGGAATATTTCA | None | None |
| Primer, reverse | GGTGTGGAAGATCCAATC | None | None |
| Probe for wild type | CCACCATGA**T**GTGCATCATT | HEX | BHQ-1 |
| Probe for p.H1047R | CCACCATGA**C**GTGCATCATT | FAM | BHQ-1 |
