# Supplementary material for: Detection of PIK3CA hotspot mutations in canine mammary tumors using droplet digital PCR: tissue validation and liquid biopsy feasibility
Source: Sci Rep. 2024 Oct 26;14:25587. doi: 10.1038/s41598-024-76820-0 (PMC11512996; doi:10.1038/s41598-024-76820-0)
Supplement: Supplementary file 3 — Supplementary Material 3 [file 41598_2024_76820_MOESM3_ESM.pptx]

## Slide 1
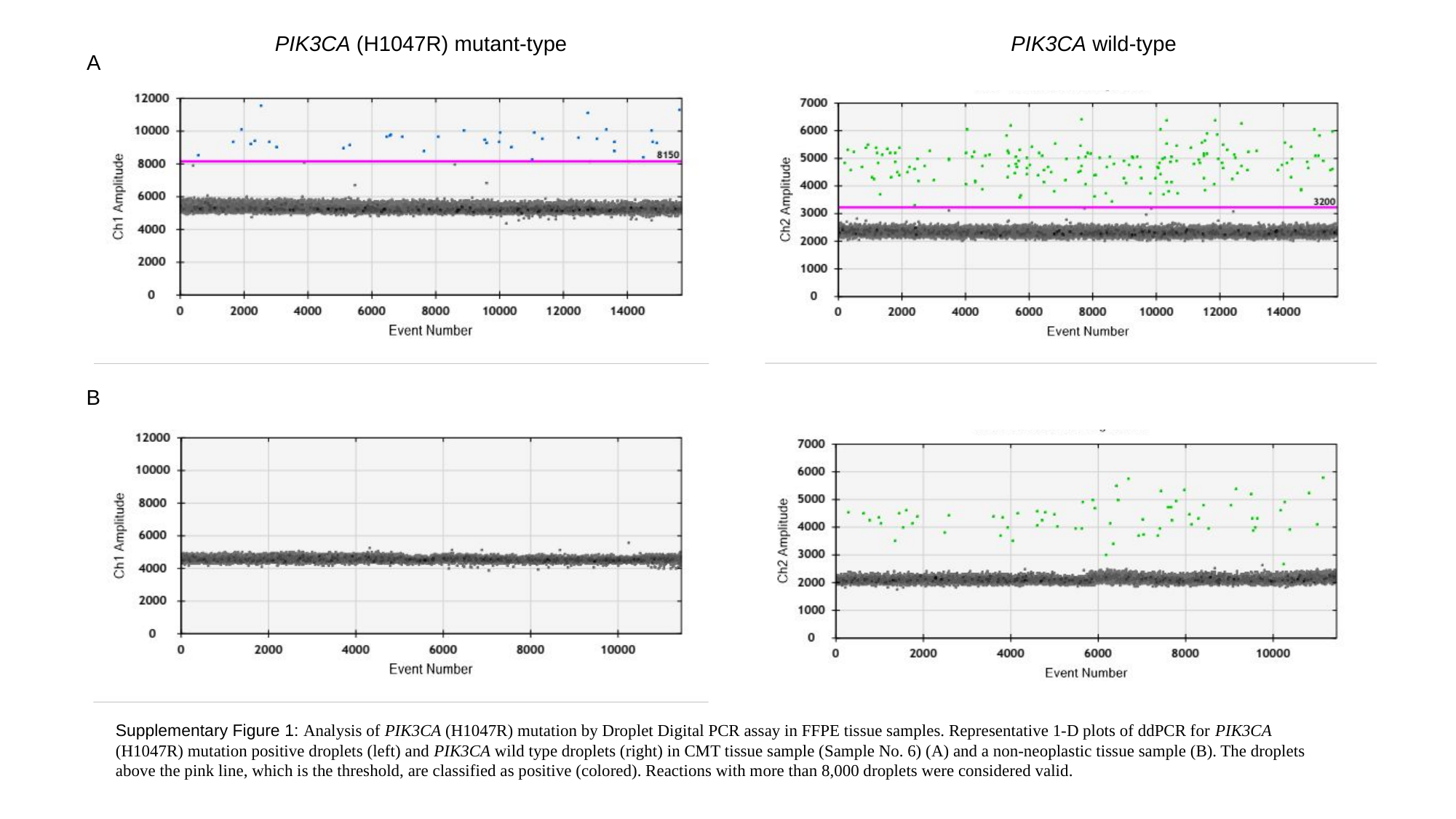

PIK3CA (H1047R) mutant-type
PIK3CA wild-type
A
B
Supplementary Figure 1: Analysis of PIK3CA (H1047R) mutation by Droplet Digital PCR assay in FFPE tissue samples. Representative 1-D plots of ddPCR for PIK3CA (H1047R) mutation positive droplets (left) and PIK3CA wild type droplets (right) in CMT tissue sample (Sample No. 6) (A) and a non-neoplastic tissue sample (B). The droplets above the pink line, which is the threshold, are classified as positive (colored). Reactions with more than 8,000 droplets were considered valid.

## Slide 2
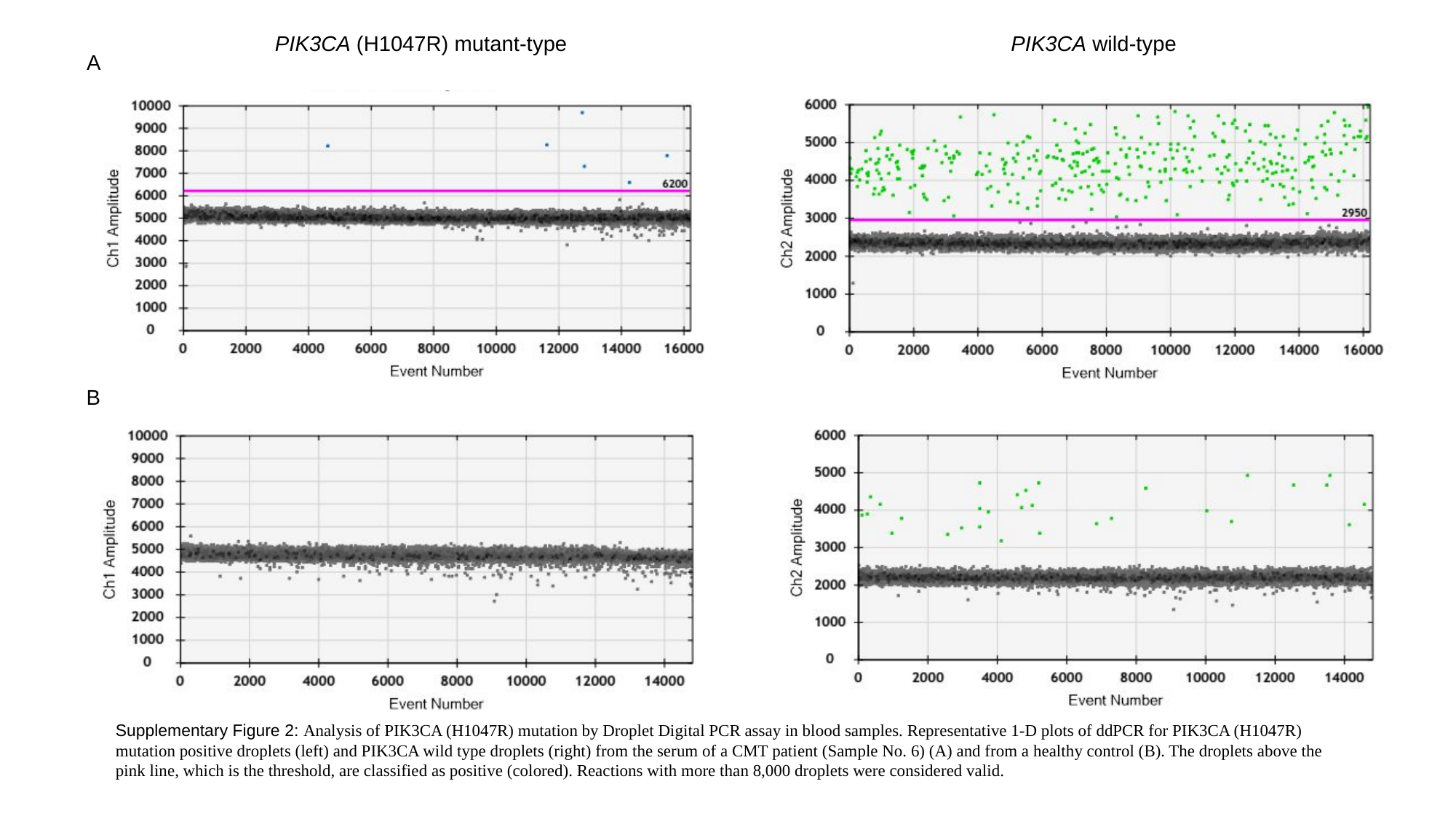

PIK3CA (H1047R) mutant-type
PIK3CA wild-type
A
B
Supplementary Figure 2: Analysis of PIK3CA (H1047R) mutation by Droplet Digital PCR assay in blood samples. Representative 1-D plots of ddPCR for PIK3CA (H1047R) mutation positive droplets (left) and PIK3CA wild type droplets (right) from the serum of a CMT patient (Sample No. 6) (A) and from a healthy control (B). The droplets above the pink line, which is the threshold, are classified as positive (colored). Reactions with more than 8,000 droplets were considered valid.
